# Incidence of cancer among residents of high temperature geothermal areas in Iceland: a census based study 1981 to 2010

**DOI:** 10.1186/1476-069X-11-73

**Published:** 2012-10-01

**Authors:** Adalbjorg Kristbjornsdottir, Vilhjalmur Rafnsson

**Affiliations:** 1 School of Health Sciences, Faculty of Medicine, University of Iceland, Reykjavik, Iceland; 2 Department of Preventive Medicine, Faculty of Medicine, University of Iceland, IS-101, Reykjavik, Iceland

**Keywords:** Breast cancer, Basal cell carcinoma of skin, Pancreatic cancer, Non-Hodgkin’s lymphoma, Radon

## Abstract

**Background:**

Residents of geothermal areas are exposed to geothermal emissions and water containing hydrogen sulphide and radon. We aim to study the association of the residence in high temperature geothermal area with the risk of cancer.

**Methods:**

This is an observational cohort study where the population of a high-temperature geothermal area (35,707 person years) was compared with the population of a cold, non-geothermal area (571,509 person years). The cohort originates from the 1981 National Census. The follow up from 1981 to 2010 was based on record linkage by personal identifier with nation-wide death and cancer registries. Through the registries it was possible to ascertain emigration and vital status and to identify the cancer cases, 95% of which had histological verification. The hazard ratio (HR) and 95% confidence intervals (CI) were estimated in Cox-model, adjusted for age, gender, education and housing.

**Results:**

Adjusted HR in the high-temperature geothermal area for all cancers was 1.22 (95% CI 1.05 to 1.42) as compared with the cold area. The HR for pancreatic cancer was 2.85 (95% CI 1.39 to 5.86), breast cancer 1.59 (95% CI 1.10 to 2.31), lymphoid and hematopoietic cancer 1.64 (95% CI 1.00 to 2.66), and non-Hodgkins lymphoma 3.25 (95% CI 1.73 to 6.07). The HR for basal cell carcinoma of the skin was 1.61 (95% CI 1.10 to 2.35). The HRs were increased for cancers of the nasal cavities, larynx, lung, prostate, thyroid gland and for soft tissue sarcoma; however the 95% CIs included unity.

**Conclusions:**

More precise information on chemical and physical exposures are needed to draw firm conclusions from the findings. The significant excess risk of breast cancer, and basal cell carcinoma of the skin, and the suggested excess risk of other radiation-sensitive cancers, calls for measurement of the content of the gas emissions and the hot water, which have been of concern in previous studies in volcanic areas. There are indications of an exposure-response relationship, as the risk was higher in comparison with the cold than with the warm reference area. Social status has been taken into account and data on reproductive factors and smoking habits show that these do not seem to explain the increased risk of cancers, however unknown confounding can not be excluded.

## Background

Through the centuries, volcanic eruptions in Iceland have now and then emitted ash and gases, which have been carried downwind to mainland Europe; and historically such events have been associated with climate change and increased mortality in England and elsewhere [1]. Recent volcanic activities in Iceland have disturbed commercial air traffic for weeks in the years 2010 and 2011 [2,3]. People living in the close vicinity of the volcano are usually those who suffer most in cases of eruption [4]. Fortunately the eruptions do not usually last for months, although it does happen. However people living on volcanic ground may experience long-term exposure to various toxic ground gas emissions, carbon dioxide (CO_2_), hydrogen sulphide (H_2_S), radon (Rn), sulphur dioxide (SO_2_), sulphuric acid (H_2_SO_4_), hydrogen chloride (HCl), and hydrogen fluoride (HF), and these are considered to pose chronic health hazards [5-7]. Several other low-dose exposures have been mentioned, among them arsenic (As), lead (Pb), and mercury (Hg) [8]. The risk of cancer among these populations has so far been the subject of only limited study and the results have been inconsistent [8-10]. In the study from Rotorua, New Zealand, Bates et al. suggested an association of nasal and lung cancer with residence in a geothermal field, and particularly exposure to H_2_S, [9] although exposure to Rn was not high, according to later estimates [7] The study from the Azores, Portugal, found an association of female breast cancer with residence on an actively degassing geothermal field, and in that study, Amaral et al. suggested that trace elements and high Rn exposure might play a role [8]. In a study from Sicily, residents of the volcanic region of Catania province seem to have higher incidence of thyroid cancer than other populations and it is mentioned that the concentration of Rn is elevated in the environment in the area [10]. However, the authors were not able to conclude on the association of Rn exposure with the risk of thyroid cancer [10].

Geothermal water and steam have been used for decades in Iceland for domestic heating, bathing and showering, and in various industries [11,12]. In the year 2000, when seismological studies were conducted, the concentration of Rn measured 1.3 Bq/l to 9 Bq/l [13] in geothermal hot water from drilled wells. Radon gas and its progeny are the major contributors to radiation exposure of the general population and are classified as carcinogenic by the International Agency for Research of Cancer (IARC), based on an increase in lung cancer among exposed human populations [14].

The aim was to study whether residence in a high-temperature geothermal area, where inhabitants are exposed to geothermal emissions and water containing hydrogen sulphide and radon, is associated with the risk of cancer.

## Methods

Geologically, Iceland is a young island located in the North Atlantic Ocean on the boundary between the North American and Eurasian tectonic plates. These two plates are moving apart at a rate of about 2 cm per year and Iceland is an anomalous part of the ridge where deep mantle material wells up and creates a hot spot of unusually great volcanic productivity and several geothermal fields [11,12]. Iceland has a homogenous Caucasian population that grew from 229,000 in 1981 to 318,000 in 2010, the period that the study spanned [15].

This is a population-based observational study. The source of data for the cohort was the 1981 National Census in Iceland, kept at Statistics Iceland. In the census, each individual is filed under a personal identification number that is allocated to individuals at birth. The census included information on gender, age, residency, education, and the type of residence. The cohort for this study was confined to people aged 5 to 65 years at the time of the census. The personal identification numbers were used in record linkage with the National Registry to obtain information, where applicable, on the date of emigration and with the National Cause-of-Death Registry to obtain information on vital status and, where applicable, the date of death. Both these registries are kept at Statistics Iceland. In this way, it was possible to ascertain the vital and emigration status for the entire cohort. Thus it was possible to define person years at risk for each individual. Those who emigrated could not be followed up in the cancer registry after the date of emigration, even in cases where they subsequently returned.

The Icelandic Cancer Registry, established in 1955, is a nation-wide registry of all cases of cancer. The registry has virtually complete coverage and over 95% of the diagnoses are histologically confirmed [16]. The topography codes used during the study period were according to ICD-7, ICD-9, and ICD-10; however they were standardized by the registry to ICD-10, and the morphology was registered according to ICD-03. Basal cell carcinoma (BCC) has been registered since 1981 in a special file at the cancer registry. It is not counted with the overall cancers and these cases are analysed separately. The computer file of individuals in the census was linked to the Icelandic Cancer Registry by their personal identification numbers. Thus, we were able to establish whether these individuals had cancer, and if so, to identify the cancer site, morphology, and date of diagnosis.

The four-digit community code in the census was used to identify the populations living in two communities located in high-temperature geothermal areas. The first of these communities is a small town in southern Iceland (Hveragerdi), where geothermal hot water has been used since 1950 for heating greenhouses and for domestical heating, laundry, bathing, showering, and washing dishes. Geothermal hot water in Iceland is not used as drinking water, as it is unpalatable and foul smelling because of the gas and mineral content, and there is an abundance of other water sources available. The area surrounding Hveragerdi forms part of the Hengill central volcano and there are many hot springs, fumaroles and erupting geysers in the town. The second community is smaller and consists of a small town and agricultural district (Skutustadahreppur, Myvatn), located in the north-eastern part of Iceland, where geothermal hot water has been used since 1967 in industry and for domestic heating, laundry, bathing, showering, and washing dishes. This community is on the edge of the Krafla volcano. Fumes from geothermal activities are frequently seen in these communities and the rotten egg odour of hydrogen sulphide is often perceived. Both these communities are inland regions, unlike most communities in Iceland that are located in coastal regions. The geothermal field in these communities belongs to the high-temperature geothermal areas where the underground temperature at 1,000 m depth is above 150°C, and the bedrock is less than 0.8 million years old, according to descriptions of the volcanic and geothermal zones in Iceland [11,12].

**Figure 1:**
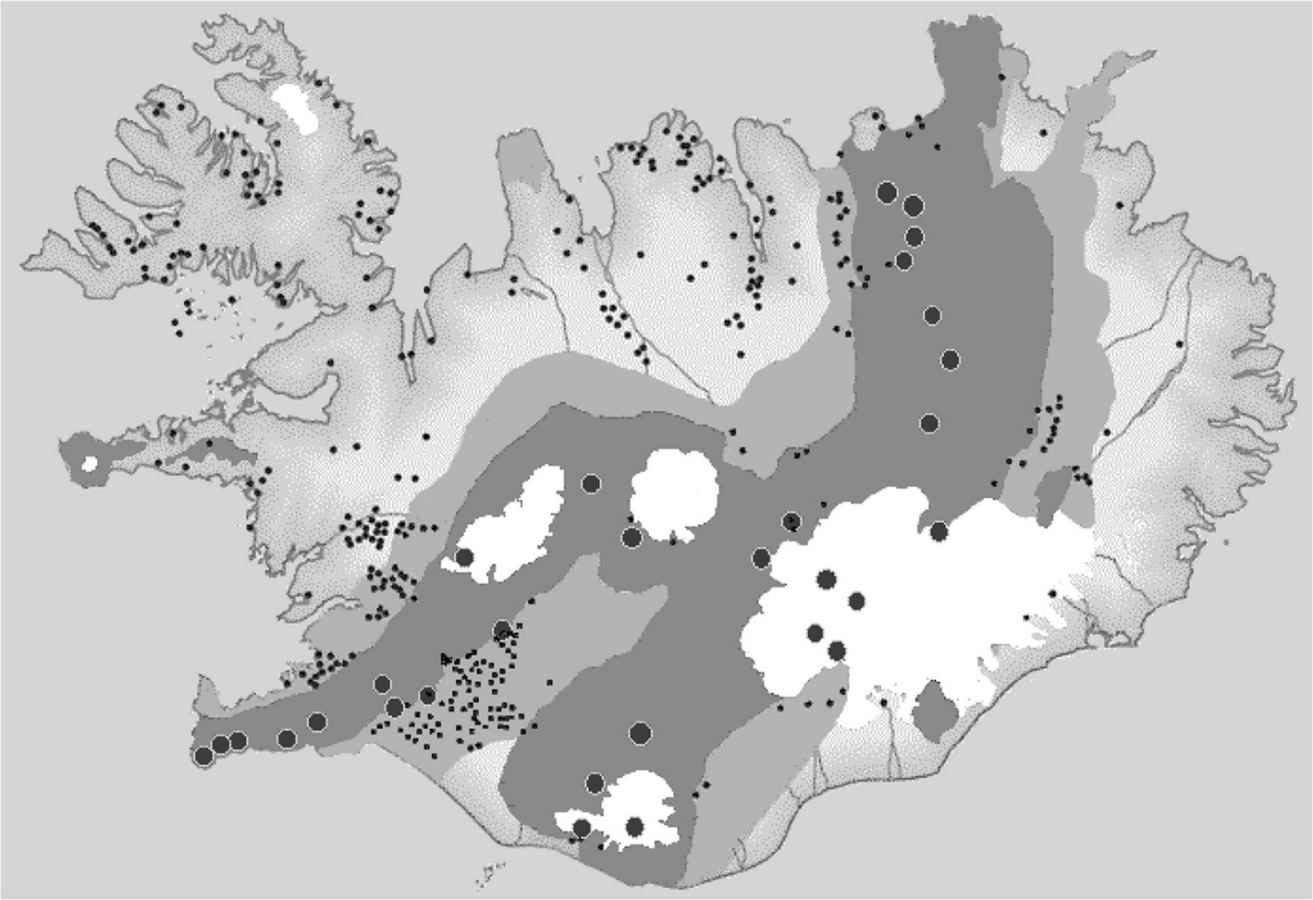
**Geological map of Iceland, showing the distribution of natural geothermal activity and the age of bedrock. ** Modified with permission from National Energy Authority. Small circles low temperature geothermal field, large circles high temperature geothermal field, age of bedrock: < 0.8, to gray 0.8-3.3, to light gray 3.3-15, and white glaciers.

**Figure 2:**
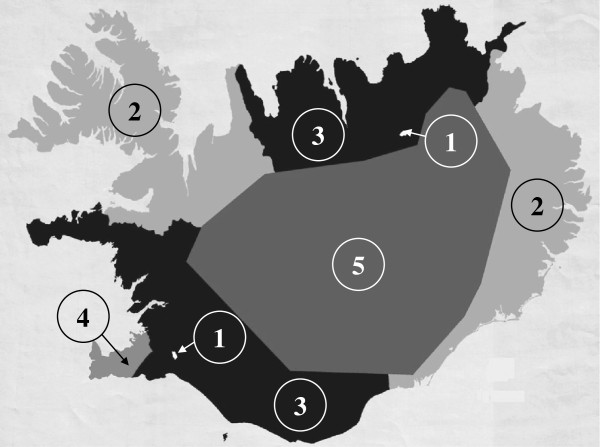
**Map of Iceland, showing the study areas according to the community codes: 1) High-temperature geothermal area. ** 2) Cold reference area. 3) Warm reference area. 4) Capital and South West area. 5) Uninhabited area. Modified with permission from National Land Survey of Iceland.

The two comparison populations, classified according to the community codes in the census, included residents of communities other than these high-temperature geothermal areas. The first of these comparison populations included residents living well outside of the volcanic zone, in what we call the cold reference area, where the bedrock is more than 3.3 million years old and the underground temperature at 1,000 m depth is below 150°C [11,12]. The population of the cold reference area is considered the main comparison population in the study. The second comparison population included those living within the volcanic zone, where the bedrock dates from different periods, [11,12] referred to here as the warm reference area. The people in the warm reference area may or may not be living in the vicinity of geothermal fields, as these are spread out over the whole country. However, the community codes did not allow for differentiation between those exposed to high-temperature geothermal fields and those who are not, so this population is considered to have a mixed exposure. The rest of population living in the capital Reykjavik and in the south-west peninsula of Reykjanes were excluded from the study. The reason were that according to the cancer registry, the capital and south-west area has had higher cancer incidence than other parts of the country for decades [16]. The geology and the location of the areas are shown in Figures 1 and 2.

The follow up started at the day of the census, 31 January 1981, and concluded at the date of emigration, or of death, or the date of diagnosis of cancer, or 31 December 2010 (the end of the follow up period), whichever occurred first. The dependent variables for this study were the incidence of first cancer occurring 31 January 1981 to 31 December 2010. The Cox proportional hazard model was used to estimate hazard ratio (HR) and 95% confidence intervals (95% CI) for all cancers and selected cancer sites in time-dependent analyses [17]. Gender was introduced as a dichotomous variable, and age as a continuous variable in years. Educational level (basic, medium and academic education), was introduced as a categorical variable according to the previous classification in a census study [18] with an additional two categories: one, unclassified for people under 20 years of age, who had not yet attained their full education level, and one missing educational information for individuals who did not indicate their education in the census. Type of residence, single-family house or other type of residence, was introduced as a dichotomous variable. According to Statistics Iceland, we divided the whole population into those living in the capital region, other urban regions and rural regions [15]. The exposed population living in the high-temperature geothermal areas was compared with the other two populations (warm reference area and cold reference area) in separate analyses. We did several calculations in the model: crude comparison without any adjustments, comparison with adjustment for age and gender only, and with adjustment for age, gender, educational level, and lodging. These three models, as well as the calculations done by introducing age stratified in 10-year age groups, had nearly identical results. Only the results with all the adjustments are presented here. All cancer sites with any cancer case are shown in the tables for completeness, and sites with no case are not shown. In cases where there were few instances of the cancer site, the confidence intervals were computed by a bootstrap resampling method. Separate analyses were done after dividing the material into gender and groups of individuals who were 20 years of age or older at the time of the census and those who were under 20 years of age, in order to investigate possible bias from childhood cancer. The statistical analyses were performed using the PASW (SPSS) software version 18, STATA, and Microsoft Excel 2007.

The National Bioethics Committee (VSNb2010060005/03.1) and the Data Protection Commission (2010060524ÞPJ/--) approved the study.

## Results

The number of individuals aged 5 to 65 years included in the census was 184,114, and the number of persons in the same age range in the National Registry was 185,610 at the time of the census, thus 99.2% were included in the census.

The number of individuals in the study was 74,806 and altogether there were 1901,786 person-years in the study. The average follow-up was 24.9 years. A total of 7,689 (4,039 men and 3,650 women) cases of first cancer were identified through the cancer registry, and there were 1,028 cases of first BCC (463 men and 565 women). During the follow up 10,570 individuals (5,599 men and 4,971 women) had emigrated and 6,458 (4,040 men and 2,418 women) had died. At the end of the study on 31 December 2010 there were 50,089 (25,449 men and 24,640 women) individuals alive without cancer.

Table 1 shows the characteristics of the cohort according to the 1981 census. The proportion of men was 52% and of women, 48%. The high-temperature geothermal areas were exclusively rural regions, the cold reference area was a mixture of other urban and rural regions, and the warm reference area was 40% other urban and 60% rural regions. As these variables have extreme and different distribution among the areas, it was not possible to enter them into the Cox-model; however, the cold area resembles the high-temperature geothermal areas with regard to these variables.

**Table 1:** Baseline characteristics in the high-temperature geothermal areas and different reference areas according to census 1981

	**Geothermal areas N (%)**	**Warm reference area N (%)**	**Cold reference area N (%)**
Number of people	1,497 (100)	50,878 (100)	22,431 (100)
**Gender**			
Men	767 (51.2)	26,431 (51.9)	11,929 (53.2)
Woman	730 (48.8)	24,447 (48.1)	10,502 (46.8)
**Age, year**			
Mean ± SD	29.69 ± 17.22	28.12 ± 16.28	28.06 ± 16.20
Median, IQR (0.25 ; 0.75)	27 (15 ; 43)	25 (15 ; 40)	25 (15 ; 39)
**Education**			
Basic education	368 (24.6)	13,831 (27.2)	6,565 (29.3)
Medium education	428 (28.6)	13,414 (26.4)	5,665 (25.3)
Academic education	140 (9.4)	3,653 (7.2)	1,428 (6.4)
Unclassified	535 (35.7)	19,404 (38.1)	8,481 (37.7)
Missing	36 (1.7)	576 (1.1)	292 (1.3)
**Housing**			
Single family home	1,205 (80.5)	33,761 (66.4)	17,343 (77.3)
Other type of house	292 (19.5)	17,117 (33.6)	5,088 (22.7)
**Region**			
Capital region	0	0	0
Other urban regions	0	20,958 (41.2)	4,863 (21.7)
Rural regions	1,497 (100.0)	29,920 (58.8)	17,568 (78.3)

*Abbreviations*: *SD* standard deviation, *IQR* interquartile range.

Table 2 shows the number of all cancers, and selected cancer sites in the high-temperature geothermal areas, and the HR and 95% CI. During the follow up, 184 cases of cancers were diagnosed among men and women in the high-temperature geothermal areas and the HRs for all sites were 1.16 (95% CI 1.00 to 1.34) and 1.22 (95% CI 1.05 to 1.42) compared with the warm reference area and the cold reference area respectively. The HRs for pancreatic cancer were 2.57 (95% CI 1.30 to 5.07), and 2.85 (95% CI 1.39 to 5.86) compared with the warm reference area and the cold reference area respectively. The HRs for bone cancer were 3.56 (95% CI 0.83 to 15.27), and 5.80 (95% CI 1.11 to 30.32) compared with the warm reference area and the cold reference area respectively, based on two cases; however, the CIs according to the bootstrap method were wide and included unity. The HRs for breast cancer were 1.43 (95% CI 1.00 to 2.05), and 1.59 (95% CI 1.10 to 2.31) compared with the warm reference area and the cold reference area respectively. The HRs for all cancers of lymphoid and haematopoietic tissue combined were 1.53 (95% CI 0.95 to 2.46), and 1.64 (95% CI 1.00 to 2.66) compared with the warm reference area and the cold reference area respectively. The HRs for non-Hodgkins lymphoma (NHL) were 3.21 (95% CI 1.77 to 5.82), and 3.25 (95% CI 1.73 to 6.07) compared with the warm reference area and the cold reference area respectively. The HRs for several other cancer sites were increased, but these were based on few cases and with wide confidence intervals. The HRs for the 30 cases of BCC in the high-temperature geothermal areas were 1.37 (95% CI 0.95 to 1.97), and 1.61 (95% CI 1.10 to 2.35) compared with the warm reference area and the cold reference area respectively, shown in the lowest row of Table 2.

**Table 2:** Number of all cancers and selected cancer sites among men and women combined in the high-temperature geothermal areas, hazard ratio (HR), 95% confidence intervals (CI) according to compared with the populations in warm reference area and cold reference area, adjusted for age, gender, education, and type of housing

**Cancers (ICD-10)**	**Geothermal areas**	**Warm reference area**		**Cold reference area**	
	**p-yr 35,707**	**p-yr 1294,570**		**p-yr 571,509**	
	**No of cancers**	**HR**	**95%CI**	**HR**	**95%CI**
All sites (C00-C97 and D45-D47)	184	**1.16**	**1.00 to 1.34**	**1.22**	**1.05 to 1.42**
Lip, oral cavity, and pharynx (C00-C14)	2	0.64	0.16 to 2.60	0.81	0.20 to 3.38
Oesophagus (C15)	1	0.50	0.07 to 3.58	0.58	0.08 to 4.20
Stomach (C16)	7	1.13	0.53 to 2.41	0.99	0.46 to 2.14
Colon, rectum, and anus (C18-C21)	16	1.13	0.69 to 1.86	1.17	0.70 to 1.94
Bile and liver (C22-C24)	2	0.95	0.23 to 3.88	1.02	0.24 to 4.29
Pancreas (C25)	9	**2.57**	**1.30 to 5.07**	**2.85**	**1.39 to 5.86**
Nasal cavity and middle ear (C30)	1	3.32	0.42 to 26.32	2.58	0.30 to 22.33
Larynx (C32)	2	2.21	0.53 to 9.30	3.04	0.66 to 13.98
Lung and bronchus (C33-C34)	20	1.24	0.80 to 1.95	1.11	0.70 to 1.75
Bone (C40-C41)	2	3.56	0.83 to 15.27*	**5.80**	1.11 to 30.32*
Melanoma (C43)	2	0.51	0.13 to 2.04	0.62	0.15 to 2.56
Other cancer of skin (C44)	4	0.84	0.31 to 2.27	1.01	0.37 to 2.79
Soft tissue sarcoma (C49)	2	1.86	0.45 to 7.78	1.97	0.45 to 8.66
Breast (C50)	31	**1.43**	**1.00 to 2.05**	**1.59**	**1.10 to 2.31**
Vulva (C51)	1	4.03	0.50 to 32.36	2.96	0.34 to 25.58
Cervix uteri (C53)	2	0.85	0.21 to 3.45	1.04	0.25 to 4.37
Uterus (C54-C55)	3	0.82	0.26 to 2.60	0.88	0.27 to 2.82
Ovary (C56-C57)	5	1.25	0.51 to 3.05	1.25	0.50 to 3.12
Prostate (C61)	29	1.16	0.80 to 1.68	1.37	0.93 to 2.00
Kidney (C64-C66)	5	0.67	0.28 to 1.62	0.83	0.34 to 2.04
Bladder (C67)	8	1.12	0.56 to 2.28	1.02	0.50 to 2.10
Brain and central nervous system (C70-C72, C75.1 and C75.3)	5	0.82	0.34 to 2.00	0.90	0.37 to 2.23
Thyroid gland (C73)	6	1.51	0.66 to 3.42	1.51	0.65 to 3.50
Cancer without specification of site (C80)	1	0.32	0.04 to 2.26	0.28	0.04 to 2.05
Lymphoid and haematopoietic tissue (C81-C96 and D45-D47)	18	1.53	0.95 to 2.46	**1.64**	**1.00 to 2.66**
Hodgkins lymphoma (C81)	1	1.03	0.14 to 7.56	1.50	0.19 to 11.61
Non-Hodgkins lymphoma (C82-C85)	12	**3.21**	**1.77 to 5.82**	**3.25**	**1.73 to 6.07**
Immunoproliferative diseases (C88)	1	1.31	0.18 to 9.76	2.00	0.24 to 16.40
Leukaemia (C91-C95 and D45-D47)	4	1.07	0.39 to 2.89	1.07	0.39 to 2.95
Chronic lymphocytic leukaemia (CLL)(C91.1)	1	0.76	0.10 to 5.54	0.70	0.09 to 5.24
Non-CLL (C91-C95 and D45-D47, except C91.1)	3	1.23	0.39 to 3.90	1.30	0.40 to 4.22
**Not included in all cancers**	p-yr 36,606	p-yr 1320,220		p-yr 581,772	
Basal cell carcinoma of the skin	30	1.37	0.95 to 1.97	**1.61**	**1.10 to 2.35**

*Abbreviation*: *p-yr*, person years.

Statistically significant HRs are bolded.

* 95% *CI* computed with bootstrap method were wide and included unity.

Among men, 90 cases of cancer were in the high-temperature geothermal areas. Table 3 shows the number of all cancers and selected cancer sites, and the HR and 95% CI. The HRs for pancreatic cancer were 2.52 (95% CI 1.01 to 6.28), and 3.66 (95% CI 1.37 to 9.82) compared with the warm reference area and the cold reference area respectively. The HRs for NHL were 3.12 (95% CI 1.43 to 6.78), and 2.58 (95% CI 1.16 to 5.78) compared with the warm reference area and the cold reference area respectively. The HRs for the 15 cases of BCC were 1.52 (95% CI 0.90 to 2.55), and 1.78 (95% CI 1.04 to 3.05) compared with the warm reference area and the cold reference area respectively.

**Table 3:** Number of all cancers and selected cancer sites among men only in the high - temperature geothermal areas, hazard ratio (HR), 95% confidence intervals (CI) according to comparison with the populations in warm reference area and cold reference area, adjusted for age, gender, education, and type of housing

**Cancers (ICD-10)**	**Geothermal areas**	**Warm reference area**		**Cold reference area**	
	**p-yr 18,181**	**p-yr 667,069**		**p-yr 300,297**	
	**No of cancer**	**HR**	**95%CI**	**HR**	**95%CI**
All sites (C00-C97 and D45-D47)	90	1.06	0.86 to 1.30	1.14	0.92 to 1.42
Lip, oral cavity, and pharynx (C00-C14)	2	0.99	0.24 to 4.03	1.14	0.27 to 4.82
Oesophagus (C15)	1	0.63	0.09 to 4.55	0.91	0.12 to 6.86
Stomach (C16)	4	1.02	0.38 to 2.77	0.78	0.29 to 2.14
Colon, rectum, and anus (C18-C21)	9	1.12	0.58 to 2.18	1.14	0.58 to 2.25
Bile and liver (C22-C24)	1	0.68	0.09 to 4.93	1.37	0.17 to 10.83
Pancreas (C25)	5	**2.52**	**1.01 to 6.28**	**3.66**	**1.37 to 9.82**
Nasal cavity and middle ear (C30)	1	6.46	0.75 to 55.75	13.08	0.79 to 215.51
Larynx (C32)	2	2.78	0.65 to 11.81	4.30	0.89 to 20.84
Lung and bronchus (C33-C34)	9	1.00	0.52 to 1.94	0.95	0.48 to 1.86
Other cancer of skin (C44)	3	1.12	0.35 to 3.54	1.22	0.38 to 3.98
Soft tissue sarcoma (C49)	1	1.62	0.22 to 12.13	2.52	0.30 to 21.04
Prostate (C61)	29	1.16	0.80 to 1.68	1.37	0.93 to 2.00
Kidney (C64-C66)	2	0.43	0.11 to 1.74	0.54	0.13 to 2.22
Bladder (C67)	7	1.24	0.58 to 2.65	1.11	0.52 to 2.41
Brain and central nervous system (C70-C72, C75.1 and C75.3)	2	0.65	0.16 to 2.64	0.70	0.17 to 2.89
Thyroid gland (C73)	1	0.73	0.10 to 5.30	1.08	0.14 to 8.21
Lymphoid and haematopoietic tissue (C81-C96 and D45-D47)	11	1.59	0.87 to 2.91	1.62	0.87 to 3.02
Non-Hodgkins lymphoma (C82-C85)	7	**3.12**	**1.43 to 6.78**	**2.58**	**1.16 to 5.78**
Leukaemia (C91-C95 and D45-D47)	4	1.73	0.63 to 4.75	1.68	0.60 to 4.74
Chronic lymphocytic leukaemia (CLL)(C91.1)	1	1.33	0.18 to 9.84	1.19	0.16 to 9.14
Non-CLL (C91-C95 and D45-D47, except C91.1)	3	1.95	0.61 to 6.25	2.00	0.60 to 6.66
**Not included in all cancers**	p-yr 18,463	p-yr 678,577		p-yr 305,053	
Basal cell carcinoma of the skin	15	1.52	0.90 to 2.55	**1.78**	**1.04 to 3.05**

*Abbreviation*: *p-yr*, person years.

Statistically significant HRs are bolded.

Among women, 94 cases of cancer were in the high-temperature geothermal areas and Table 4 shows the number of all cancers and selected cancer sites, and the HR and 95% CI. The HRs for all sites were 1.27 (95% CI 1.03 to 1.56), and 1.30 (95% CI 1.05 to 1.61) compared with the warm reference area and the cold reference area respectively. The HRs for bone cancers were 7.95 and 7.20 compared with the warm reference area and the cold reference area respectively, based on two cases; however the CIs according to the bootstrap method were wide and included unity. The HRs for breast cancer were 1.46 (95% CI 1.02 to 2.09), and 1.62 (95% CI 1.12 to 2.36) compared with the warm reference area and the cold reference area respectively. The HRs for NHL were 3.31 (95% CI 1.32 to 8.34), and 5.20 (95% CI 1.87 to 14.45) compared with the warm reference area and the cold reference area respectively.

**Table 4:** Number of all cancers and selected cancer sites among women only in the high - temperature geothermal areas, hazard ratio (HR), 95% confidence intervals (CI) according to comparison with the populations in warm reference area and cold reference area, adjusted for age, gender, education, and type of housing

**Cancers (ICD-10)**	**Geothermal areas**	**Warm reference area**		**Cold reference area**	
	**p-yr 17,526**	**p-yr 627,500**		**p-yr 271,213**	
	**No of cancer**	**HR**	**95%CI**	**HR**	**95%CI**
All sites (C00-C97 and D45-D47)	94	**1.27**	**1.03 to 1.56**	**1.30**	**1.05 to 1.61**
Stomach (C16)	3	1.33	0.42 to 4.22	1.55	0.46 to 5.14
Colon, rectum and anus (C18-C21)	7	1.16	0.54 to 2.46	1.24	0.57 to 2.70
Bile and liver (C22-C24)	1	1.54	0.21 to 11.56	0.82	0.11 to 6.17
Pancreas (C25)	4	2.68	0.96 to 7.45	2.26	0.78 to 6.52
Lung and bronchus (C33-C34)	11	1.56	0.85 to 2.86	1.26	0.68 to 2.33
Bone (C40-C41)	2	**7.95**	1.70 to 37.23*	**7.20**	1.30 to 39.96*
Melanoma (C43)	2	0.85	0.21 to 3.46	0.85	0.20 to 3.53
Other cancer of skin (C44)	1	0.48	0.07 to 3.43	0.66	0.09 to 4.91
Soft tissue sarcoma (C49)	1	2.21	0.29 to 16.90	1.62	0.20 to 12.90
Breast (C50)	31	**1.46**	**1.02 to 2.09**	**1.62**	**1.12 to 2.36**
Vulva (C51)	1	4.03	0.50 to 32.36	2.96	0.34 to 25.58
Cervix uteri (C53)	2	0.85	0.21 to 3.45	1.04	0.25 to 4.37
Uterus (C54-C55)	3	0.82	0.26 to 2.60	0.88	0.27 to 2.82
Ovary (C56-C57)	5	1.25	0.51 to 3.05	1.25	0.50 to 3.12
Kidney (C64-C66)	3	1.04	0.33 to 3.29	1.27	0.39 to 4.16
Bladder (C67)	1	0.67	0.09 to 4.85	0.65	0.09 to 4.85
Brain and central nervous system (C70-C72, C75.1 and C75.3)	3	1.01	0.32 to 3.20	1.11	0.34 to 3.62
Thyroid gland (C73)	5	1.92	0.78 to 4.74	1.65	0.65 to 4.18
Cancer without specification of site (C80)	1	0.55	0.08 to 4.01	0.56	0.08 to 4.11
Lymphoid and haematopoietic tissue (C81-C96 and D45-D47)	7	1.46	0.68 to 3.12	1.66	0.76 to 3.63
Hodgkins lymphoma (C81)	1	2.13	0.28 to 16.22	4.42	0.49 to 39.63
Non-Hodgkins lymphoma (C82-C85)	5	**3.31**	**1.32 to 8.34**	**5.20**	**1.87 to 14.45**
Immunoproliferative diseases (C88)	1	4.38	0.54 to 35.34	11.92	0.72 to 197.59
**Not included in all cancers**	p-yr 18,143	p-yr 641,643		p-yr 276,719	
Basal cell carcinoma of the skin	15	1.24	0.74 to 2.08	1.45	0.85 to 2.47

*Abbreviation*: *p-yr*, person years.

* 95% CI computed with bootstrap method were wide and included unity.

Statistically significant HRs are bolded.

When confined to individuals 20 years of age and older and excluding those with missing information on education in the 1981 census, there were altogether 172 cancer cases in the high-temperature geothermal areas. In this older part of the cohort, the comparison of the high-temperature areas with the cold reference area yielded similar HRs as in the total exposed cohort. The HRs were somewhat lower and the 95% confidence intervals were a little wider, but the intervals were still not including unity for all cancers, pancreatic cancer, breast cancer and NHL. The HR for BCC was 1.52 (95% CI 1.01 to 2.73) based on 26 cases.

In the analysis of those who were under 20 years of age at the census, there were 12 cancers, three among men and nine among women, in the high-temperature geothermal areas. The mean age in this group of cancer cases was 16 years (range 11 to 19 years) at the 1981 census, and the mean age at diagnosis of the cancer was 33.4 years (range 18 to 45 years). The HR for breast cancer was 2.99 (95% CI 1.03 to 8.66), based on four cases, when comparing this younger part of the exposed cohort with the cold reference area. For other cancer sites there were fewer cases. The HR for BCC was 2.70 (95% CI 0.94 to 7.73) based on four cases.

## Discussion

This study based on 184 cancer cases in high-temperature geothermal areas showed an excess for all cancers as compared with the reference areas. There is evidence of an exposure-response relationship, as the HRs were higher in the comparison with the cold reference area than with the warm reference area. The most significant results are the excess of BCC in the total cohort based on 30 cases, and the excess of breast cancer and NHL among women and the excess of NHL and pancreatic cancer among men. Many of these cancer sites, which in the present study are found increased were not included in previous studies of the populations of geothermal areas [8-10]. However, breast cancer was found in excess among the female population of Furnas, Azores [8] and in that study a high rate for cancer of the lip, oral cavity and pharynx was found, although it was based on few cases.

### Pancreatic cancer

There was a high rate of pancreatic cancer in the total exposed cohort and among men and a non-significant elevated rate among women; male gender is one of the risk factors for pancreatic cancer. The most important external risk factor for this malignancy, smoking, is not known on an individual basis, but information on smoking in these populations is accessible from the annual surveys of the Public Health Institute of Iceland [19] from the year 1989 to 2010. These data show that the number of smokers has been declining from 31.0% to 14.2% in the population over the period, and the number of smokers has been similar in the capital area and in the rest of the country for decades, so smoking habits are not likely to be a confounder. Supporting this view is the fact that the lung cancer rate was not statistically increased in the exposed cohort. There is no obvious connection of the geothermal field pollution and pancreatic cancer, as the carcinogenic effect of Rn has in most of the studies been related to lung cancer. However, in a collaborative analysis of 11 studies of Rn-exposed underground miners, Darby et al. found an O/E of 1.05, 95%CI 0.85 to 1.29, for pancreas cancer mortality, and that mortality for pancreatic cancer was significantly related to cumulative exposure [20]. In the conclusion of that study, this relation was dismissed, despite the fact that pancreatic cancer is with high mortality and therefore suitable for a mortality study, as compared to cancers with better prognosis. The Rn exposure of miners is higher than Rn exposure in studies on residents.

### Breast cancer

As indicated previously, the elevated incidence of breast cancer is in line with the finding in the much smaller study on the population in Furnas, [8] and the authors concluded that the increased risk of breast cancer may be partially explained by the gas emission, trace elements and Rn exposure. Besides the Portuguese study [8] there is scanty literature on the association of exposure to Rn and cancer among women: there were no female workers in the mining populations [21] and the case–control studies on residential exposure to Rn did not deal with breast cancer.

Data on reproductive factors, the most important possible confounder in breast cancer studies of this design, [22] were available from Statistics Iceland [15]. Between 1991 and 1995, the fertility rate for the high-temperature geothermal areas was 2.2, for the warm reference area 2.2, and for the cold reference area 2.3. The figures for mean age at first birth were 22.6 for the high-temperature geothermal areas, 23.1 for the warm reference area, and 23.3 for the cold reference area. This information suggests that reproductive factors are not positive confounders for breast cancer in this study.

In Iceland screening with mammography have been offered to all women 40 to 69 years of age since 1987, and there are no indications of differences in participation in the screening program according to residency.

### Bone cancer

The best known etiological factors for bone cancer are ionizing radiation, radionuclides and x-ray therapy, and alkylating agents [23]. In the present study, only first cancers were included and thus therapeutic ionizing radiation and chemotherapy with alkylating agents is unlikely to be involved. The histology of bone cancers was one giant cell sarcoma and one hemangiosarcoma. This rare malignancy was not found in excess among the mining populations [20].

### NHL

NHL is heterogeneous in aetiology and is classified into many histological types and by sites of origin. Many infectious agents, immune deficiencies, autoimmunity and high doses of ionizing radiation have been associated with NHL. Furthermore, other possible risk factors are farming, pesticides, organochlorines, besides host factors such as personal and family history of cancer and certain medical conditions [24]. A detailed knowledge of these risk factors among the population of the high-temperature geothermal areas or the reference areas is not at hand; however considerable agricultural activity and greenhouses were present in one of the areas. Previous study on pesticide users in Iceland did not find increased incidence of NHL [25].

NHL has not been associated with Rn exposure in miners [20].

### Lung cancer

In the study from Rotorua the cancer concern in relation to the geothermal gas emissions were foremost cancers of the respiratory system [9] with some indication of elevated risk for lung cancer. In the present study the incidence of lung cancer was increased and more so among women, however, the confidence interval included unity. There is a general agreement on the interaction between Rn exposure and tobacco smoking on one hand and lung cancer risk on the other [14]. Breaking the smoking information from the Public Health Institute of Iceland [19] down on the geographical areas in this study showed the proportion of never smokers were fairly equally distributed among the studied populations, although highest in the high-temperature geothermal areas: 47.6% (based on 479 answers), warm areas: 45.4% (based on 16,187 answers), and cold areas 46.8% (based on 5,524 answers). Thus it seems unlikely that smoking habits were confounding the non-statistical significant increased risk for lung cancer.

### BCC

Exposures to Rn and alpha radiation have previously been associated with BCC in a study of uranium miners [26]. In that study, surface contamination of the skin by Rn and its progeny was considered of importance, as the basal cell layer of the skin lies within the range of the alpha particles [26]. Ionizing radiation exposure and ultraviolet radiation from the sun are well known causes of BCC, and the interaction of these factors has been debated and partially rejected [27]. A recent ecological study in South West England showed an association of residential exposure to Rn and risk of squamous cell carcinoma, [28] and a previous study in the same setting also indicated an association of residential exposure to Rn and non-melanoma skin cancers. In that study, basal cell carcinoma was included among the non-melanoma skin cancers [29]. Arsenic in the water is unlikely to be a positive confounder for skin cancer in the present study, as geothermal hot water was not used as drinking water. Excessive exposure to ultraviolet radiation is also not likely to be a confounder for the BCC in this study, as there is no corresponding increase in the rates of malignant melanoma or other skin cancers.

### Other cancers

Many of the rarer cancer sites had few cases; however high HRs were observed for cancers of the nasal cavities, larynx, prostate, and for soft tissue sarcoma. The standardized incidence ratio for cancer of the nasal cavities was increased among the population of Rotorua, New Zealand [9]. The rates for prostate cancer and cancer of the thyroid gland were elevated, but the 95% CI for these cancer sites included unity.

### Strength and limitations

To our knowledge, the follow up time in our cohort study is the longest of the populations in geothermal areas, thus far. The strength of the study is the use of comprehensive population registries and the universal use of personal identification numbers, which enabled accurate record linkage. For the cohort, it was thus possible to ascertain vital and emigration status through the National Registry and National Cause-of-Death Registry for all cohort members, and complete identification of cancer cases was ensured through the Icelandic Cancer Registry. The nation-wide cancer registry is virtually complete, with more than 95% of the diagnoses histologically confirmed, and the registry was used for case finding for both the exposed population and the reference populations [16].

BCC may be considered a special case, as these were all histologically verified and the incidence has increased dramatically through the years along with the incidence of malignant melanoma and other skin cancers in the Icelandic population. The increase may in part be attributed to more complete reporting to the cancer registry. In the study on skin cancer in South West England, the proportion of BCC to all non-melanoma skin cancers was 70%, [28] and the corresponding figure in the present study was 85% for the total cohort and 88% for the high-temperature geothermal areas.

The HR for all cancers combined was increased and so were HRs for several cancer sites, the rare sites showed for descriptive purposes. In no case when the HRs for certain cancer site were decreased were they followed by confidence intervals which included unity. So the whole pattern inclines towards increased risk for cancer in the high-temperature geothermal areas. Nevertheless concern may arise about a need of adjustment for multiple comparison, however, it has been maintained that these are not needed [30].

Both the present study and previous studies on populations in geothermal areas have been limited by a lack of individual exposure information on the cohort members in terms of mode and magnitude of the ground gas emission and the exact content of the hot water [8-10].

Census-based studies, including studies from Iceland, have in any case been widely used to evaluate occupational and socioeconomic determinates of cancers [18,31]. That type of study is often handicapped because of limited control on possible confounders. However, in the present study, we were able to adjust for educational level, lodging, and residential areas. On the other hand, we were only able to control indirectly for possible confounding from fertility rates, the mean age at the first birth, and smoking habits.

The excess rates of the different cancer sites found in this study seem hard to explain by single carcinogenic exposure specifically to geothermal gas emission, and that is the novelty of the study. The significant excess of BCC and breast cancer, and the suggested excess of cancer sites such as bone, nasal cavity, larynx, lung, thyroid gland, and of soft tissue sarcoma, all of these being radiation–sensitive cancers, indicate that Rn may contribute to the increased risk of cancer among the population in the high-temperature geothermal areas. According to IARC, Rn and its progeny are carcinogenic because of evidence of an increased risk for lung cancer [14] and IARC has stated that internalized radionuclides that emit alpha particles are carcinogenic to humans. The role of Rn seems not to be supported by the high rates found for pancreatic cancer and NHL, as cancers of these sites have infrequently been related to ionizing radiation. However, cytogenetic analysis in peripheral lymphocytes of persons exposed to increased levels of domestic Rn concentrations showed increased frequency of the translocation in stable cells compared with control individuals [32]. It was concluded in that study that the translocations were induced in the blood-forming tissue and then transmitted to the peripheral blood [32]. The respiratory tract has been considered the main target tissue of radon and its progeny; however, a part of the inhaled radon is absorbed into the blood and transported to all tissues of the body and deposited in higher concentrations in fatty tissues [14,32,33]. Various tissues, including bone marrow, are thus exposed to alpha particles [14].

In future studies of the geothermal areas, detailed information is needed on exposure on an individual level. These studies should also gather larger data and from different settings, and information on the length of residency in the geothermal areas should be obtained, as this can be used as a surrogate of the exposures among these populations. New studies are needed to confirm or refute the present findings.

## Conclusions

More precise information on chemical and physical exposures are needed to draw firm conclusions from the findings. The significant excess risk of breast cancer and basal cell carcinoma of the skin and the suggested excess risk of other radiation sensitive cancers, calls for measurement of the content of the gas emissions and the hot water, which have been of concern in previous studies in volcanic areas. There are indications of an exposure-response relationship, as the risk was higher in comparison with the cold than with the warm reference area. Social status has been taken into account and data on reproductive factors and smoking habits show that these do not seem to explain the increased risk of cancers, however unknown confounding can not be excluded.

## Abbreviations

BCC: Basal cell carcinoma; CI: Confidence intervals; CLL: Chronic lymphocytic leukemia, HR, Hazard ratio; IARC: International Agency for Research of Cancer; ICD-7: International classification of diseases, 7^th^ revision; ICD-9: International classification of diseases, 9^th^ revision; ICD-10: International classification of diseases, 10^th^ revision; ICD-03: International classification of diseases for oncology; IQR: Inter quartile range; NHL: Non-Hodgkin’s lymphoma; Non-CLL: Non-chronic lymphocytic leukaemia; p-yr: Person years; SD: Standard deviation.

## Competing interests

Both authors declare that they have no competing interests.

## Authors’ contributions

AK and VR designed the study, planned the analysis, drafted the article, interpreted the conclusions, and agreeing on the final version. AK and VR initiated the study and VR obtained the funding and is the guarantor. All authors read and approved the final manuscript.
